# Cardioprotective medications and the incidence of cardiovascular events in patients treated with radiotherapy: a systematic review and meta-analysis

**DOI:** 10.1186/s40959-025-00439-x

**Published:** 2026-01-08

**Authors:** Vishwa Pakeerathan, Ravi Marwah, Abdul Rahman Mohammed, Justin Smith

**Affiliations:** 1 Princess Alexandra Hospital, Brisbane, Australia; 2 School of Medicine and Dentistry, Griffith University, Gold Coast, Australia; 3 Faculty of Medicine, University of Queensland, Brisbane, Australia; 4 Sunshine Coast University Hospital, Birtinya, Australia; 5 Townsville University Hospital, Townsville, Australia; 6 College of Medicine and Dentistry, James Cook University, Townsville, Australia

## Abstract

**Purpose:**

The purpose of this systematic review and meta-analysis is to explore the utilization of cardioprotective medications in patients treated with RT and assess their impact on cardiovascular and cerebrovascular events.

**Materials/methods:**

A literature search of PubMed, Embase and Scopus was performed in March 2025. Studies of adult patients treated with RT to the head and neck or thoracic regions which investigated the effects of cardioprotective medications (defined as anti-hypertensives, lipid-lowering therapies or anti-thrombotic medications) on the incidence of cardiovascular or cerebrovascular events were eligible for inclusion. Studies that reported the proportion of patients treated with RT who were utilizing cardioprotective medications as recommended by CVD guidelines were also included. Meta-analysis was performed using R with a random effects model.

**Results:**

There were 10 retrospective studies which were eligible for inclusion. Five of the ten studies included patients with head and neck cancer only, whilst two studies included patients with lung cancer and one study included patients with breast cancer alone. Meta-analysis of three studies suggested that patients treated with RT who received statin therapy had a reduced risk of cerebrovascular events (stroke or transient ischemic attack), with a relative risk of 0.74 (95% CI 0.60-0.90). There was no difference in major adverse cardiac events (MACE) for patients treated with RT to the head and neck or thoracic regions who received statin therapy compared to those who did not (relative risk 0.99, 95% CI 0.67 to 1.46, n = 5 studies). A meta-analysis of four studies suggested that 59% (95% CI 35% to 80%) of patients treated with RT not on statin therapy had indications for commencement of these medications.

**Conclusion:**

Current evidence exploring the impact of cardioprotective medications on CVD risk in patients treated with RT is heterogenous and limited to retrospective non-randomized studies. A considerable proportion of patients undergoing RT are not being prescribed cardioprotective medications as suggested by existing CVD guidelines.

**Supplementary Information:**

The online version contains supplementary material available at 10.1186/s40959-025-00439-x.

## Introduction

Approximately 30–50% of all cancer patients require radiation therapy as part of their management [1]. However, radiotherapy increases the risk of cardiovascular and cerebrovascular disease (CVD) amongst cancer survivors [2], particularly those with head and neck cancer [3], lung cancer [4], and esophageal cancer [5]. Proposed pathogenic mechanisms include accelerated endothelial damage and atherosclerosis, as well as increased inflammation and fibrosis of myocardial, pericardial, valvular and conduction tissues [6]. 

While statin therapy has been associated with reduced all-cause mortality, cardiovascular death, and incidence of myocardial infarction in the general population [7, 8], the prescription of statins is primarily guided by traditional CVD risk stratification scores including the Framingham Risk Score [9]. Importantly, prior research suggests that patients treated with radiotherapy are at higher risk of CVD than indicated by these scores [10]. Therefore, there are concerns that cardioprotective medications including statins are being underutilized in this population with an increased CVD risk. Notably, a recent prospective study found that early cardio-oncology review in patients treated with high-dose thoracic radiotherapy resulted in a change in clinical management in more than 60% of patients, predominantly in the form of statin therapy initiation or intensification [11]. 

The aim of this review was to evaluate cardioprotective pharmacotherapies, including lipid-lowering therapy (statins), antihypertensives, and antithrombotic agents in patients treated with radiotherapy, and to assess their impacts on cardiovascular and cerebrovascular outcomes, as well as to quantify the uptake of cardioprotective medications in this group of patients.

## Methods

This systematic review and meta-analysis was performed as per PRISMA guidelines.

### Eligibility criteria

Studies that met the criteria below were included.

### Inclusion criteria

- Included patients who have previously received external beam radiation therapy for cancer in the head and neck or thoracic regions (including mediastinum/breast/esophagus/lung cancers).
- Reports the incidence of cardiovascular events in a group that received radiotherapy and a group that did not, or the incidence of patients treated with RT who were prescribed cardioprotective medications.
- Cardiovascular events defined as ischemic stroke, transient ischemic attack (TIA) or myocardial infarction.
- Cardioprotective medications defined as anti-hypertensives, lipid lowering therapies or anti-thrombotic medications.

### Exclusion criteria

- Studies with a pediatric patient population.
- Included patients with haematological malignancies.
- Pre-clinical studies.

### Search strategy

A literature search was performed in March 2025 on PubMed, Scopus and Embase. The PubMed search strategy is demonstrated below, with appropriate adjustments made for Scopus and Embase (Supplementary 1). The databases were searched from inception to March 2025.

(“cancer” OR “malignancy” OR “carcinoma” OR “neoplasm” OR “tumour” OR “tumor”) AND (“radiation therapy” OR “radiotherapy” OR “irradiation”) AND (“statin” OR “lipid lowering” OR “cholesterol lowering” OR “antihypertensive” OR “anti-hypertensive” OR “blood pressure medication” OR “antithrombotic” OR “anti-thrombotic” OR “antiplatelet” OR “anti-platelet” OR “aspirin” OR “cardiovascular medication”).

### Study selection process

Initial title and abstract screening were performed by the first author and relevant articles were identified for full text review. These manuscripts were then screened independently by two authors (VP and JS) to assess eligibility for inclusion.

### Data extraction

One author (VP) extracted relevant data from included manuscripts. This data was checked for accuracy by a second author (RM). Extracted data included study methodology, sample size, study outcomes and participant characteristics.

### Risk of bias assessment

Eligible studies were assessed using the Newcastle Ottawa for cohort studies. The checklist scored eight items as responses, ‘yes’, ‘no’ or ‘unclear’. A maximum score of nine indicated that all criteria were addressed in the study. This process was conducted independently by two authors (VP and RM).

### Statistical analysis

A meta-analysis was performed if three or more studies reported the same outcome. Meta-analyses were conducted using R version 4.3.1 and a random effects model was used. Statistical significance was defined as *p* < 0.05 and study heterogeneity was assessed using I^2^ values. A meta-analysis of incidence rates was performed to compare the risk of cerebrovascular and cardiovascular events between the two groups (RT and control groups). Cerebrovascular events were defined as ischemic stroke alone, with TIA events excluded for the purposes of pooled meta-analysis. Major adverse cardiovascular events (MACE) were defined as a pooled measure of any cardiovascular (e.g. myocardial infarction, atrial fibrillation, heart failure) and cerebrovascular events (stroke or TIA). Person years were used to assess the follow-up time for each study. If a study did not report follow up in person years then this was estimated using median follow up time. If median follow up time was not reported for the two groups separately it was assumed to be the same follow up time in both groups. A meta-analysis of proportions was used to estimate the percentage of patients treated with RT who were not prescribed cardioprotective medications but had indications to commence them.

## Results

### Study selection

**Fig. 1:**
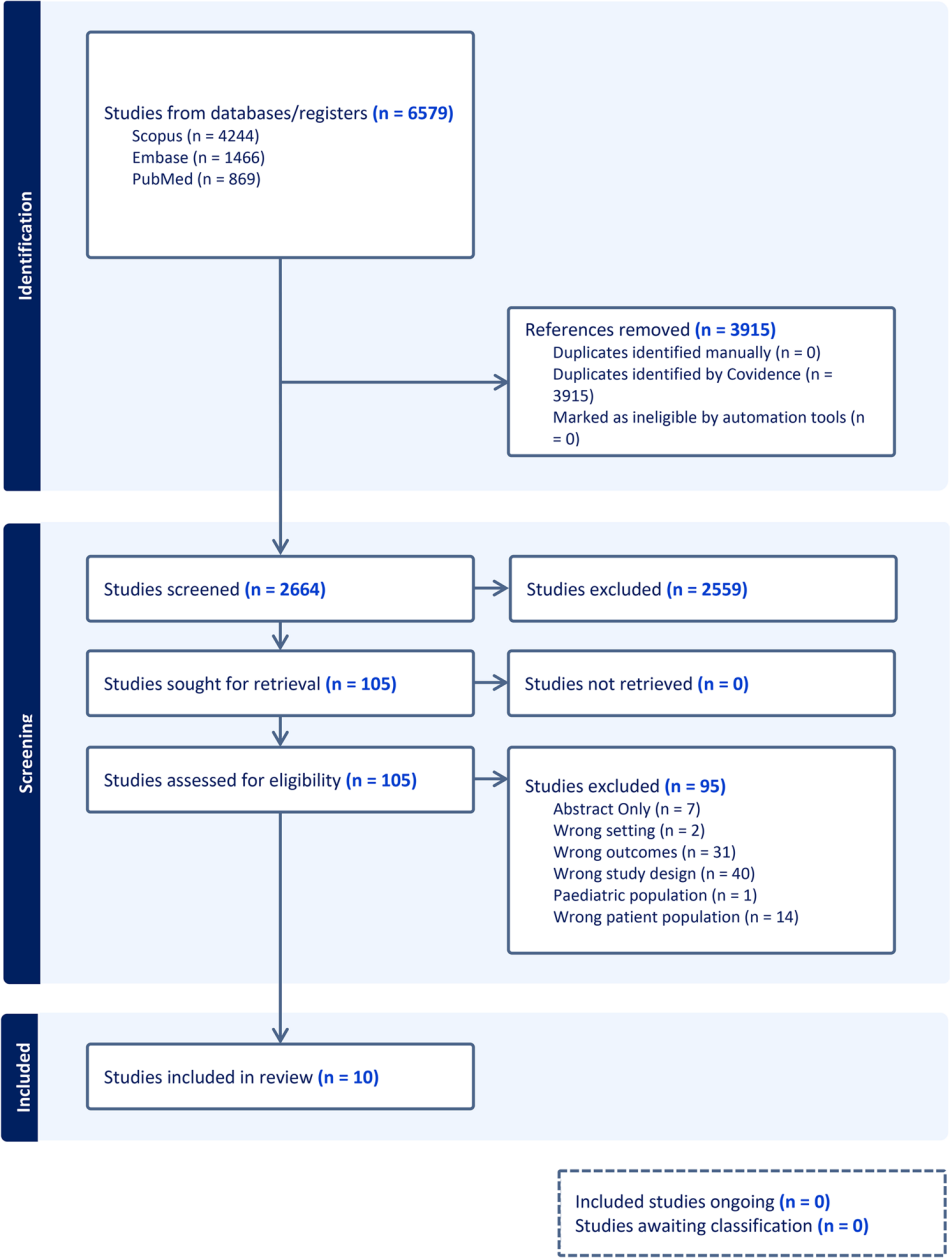
PRISMA flow diagram

There were 2664 articles screened by title and abstract (Fig. 1). 105 articles were then screened in full text, and 10 studies were deemed to meet eligibility criteria. There were 95 studies excluded in the full text screening, with 40 because of wrong study design, 31 due to wrong outcomes and 15 had the incorrect patient population. Seven were abstract only and 2 were deemed to be in the wrong setting.

### Study characteristics

Study characteristics for the 10 included studies are summarized in Table 1. There was significant variability within the included studies, with varying patient populations, tumor subsites and outcome measures. There were five studies which included patients with head and neck cancer only [12–16], two studies reported outcomes in lung cancer alone [17, 18], whilst one study included patients with breast cancer [19], one with esophageal cancer [16] and one with patients receiving thoracic and/or head and neck RT [12–16, 20]. Although the eligibility criteria included lipid-lowering, antihypertensive, and antithrombotic therapies, the majority of eligible studies assessed statin exposure, with nine of the ten studies exploring statin therapy use during or after RT. Conversely, there were only three studies which explored the effectiveness of anti-thrombotic treatment (Table 2).

**Table 1:** Study characteristics

Author (Year)	Country	Study design	Population	Sample size	Cardioprotective medication	Study objectives	Outcome measures	Events in cardioprotective group	Events in non-cardioprotective group	Median follow-Up	Proportion of patients not receiving cardioprotective mediations with indications to commence	Comments
Okoye (2016) [21]	USA	Retrospective cohort	HNC	115	Statins and anti-thrombotic (aspirin)	To quantify baseline CVD risk and assess use of preventive cardioprotective medications	Proportion of eligible patients not prescribed cardioprotective medications	N/A	N/A	2 years	38/115 (33%) of patients not receiving statins as recommended by AHA guidelines	
Hsu (2016) [14]	Taiwan	Retrospective cohort	HNC	37,638	Oral Anti-thrombotic Therapy (antiplatelet or anticoagulants) *Use defined as at least one prescription within 30 days of RT start date*	Evaluate efficacy and safety of oral anti-thrombotic therapy for primary stroke prevention in HNC patients after RT.	Ischemic stroke or TIAOther - Death and major bleeding	21 stroke/TIA(n = 815)	600 stoke/TIA(n = 36,823)	2.7 years (mean)	NR	Anti-thrombotics did not increase major bleeding risk
Addison (2017) [12]	USA	Retrospective cohort	HNC	1,011	Statins*Use defined as statin utilization at the time of RT start date*	Determine whether statin use at time of RT lowers post-RT stroke/TIA risk	Ischemic stroke and TIA	16 stroke and 1 TIA (n = 288)	73 strokes and 12 TIA(n = 723)	3.4 years	NR	Statin users had significantly lower risks of stroke/TIA post-RT (adjusted HR = 0.40, 95% CI 0.2–0.8, p = 0.01)
Boulet (2019) [20]	Canada	Retrospective cohort	HNC & Thorax	5,718	Statins*Use defined as statin prescription 1 year before RT start to event or censure*	Determine if post-RT statin use reduced vascular events (stroke, TIA, MI)	MACE	376 MACE110 strokes(n = 4166)	160 MACE56 strokes(n = 1552)	1.5 years (mean)	NR	MACE defined as MI, stroke or death caused by MI or stroke
Atkins (2021) [17]	USA	Retrospective cohort	NSCLC	748	Statins*Use defined as statin utilization at RT consultation or prior*	Assess whether statin therapy predicts overall survival after RT (and impact on cardiac events)	MACEProportion of eligible patients not prescribed cardioprotective medications	45 MACE(n = 305)	32 MACE(n = 443)	1.7 years	231/344 (67%) of patients not receiving statins had indications to commence as per Framingham risk	MACE defined as cardiac death, unstable angina, myocardial infarction, heart failure hospitalization and coronary revascularization
Alvi (2022) [13]	USA	Retrospective cohort	HNC	723	Statins*Use defined as statin utilization before or after RT*	Evaluate performance of cardiovascular risk models in predicting MACE post RT	MACEProportion of eligible patients not prescribed cardioprotective medications	53 MACE(n = 288)	139 MACE(n = 723)	6.6 years	274/723 (38%) of patients not prescribed statins had indications to commence as per USPSTF criteria and 32% based on Framingham risk	MACE defined as myocardial infarction, coronary heart disease, stroke and cardiovascular death
Walls (2023) [18]	United Kingdom	Retrospectivecohort	NSCLC	478	Statins*Use defined as statin utilization at start of RT. Statin intensity graded as low, medium or high*	Impact of statins on cardiac events and survival as well as investigating impact of statin intensity	MACEProportion of eligible patients not prescribed cardioprotective medications	50 MACE(n = 283)	29 MACE(n = 195)	1.8 years	171/195 (88%) of patients not on statins had at least one guideline indication for statin therapy	MACE defined as acute coronary syndrome, heart failure or arrhythmiaHigh intensity and medium intensity statin therapy associated with improved survival
Huang (2024) [19]	Taiwan	Retrospective cohort	Breast cancer	1,481	Statins*Use defined as patients who utilized statins during or after RT*	Determine if statin therapy reduces radiation-induced cardiotoxicity (MACE risk) after RT for breast cancer	MACE	73 MACE(n = 360)	121 MACE(n = 360)	5 years	NR	MACE defined as MI, acute coronary syndrome/ischemic heart disease, cerebrovascular accident, heart failure and cardiovascular deathPropensity score matching to balance cohortsA dose-response effect was observed
Lin (2024) [15]	Taiwan	Retrospective cohort	HNC – Nasopharyngeal only	5,022	Statins*Use defined as patients who utilized statins during or after RT*	Assess impact of statin use on ischemic stroke risk in patients receiving RT for nasopharyngeal cancer	Ischemic stroke	273 strokes(n = 2,515)	332 strokes (n = 2,507)	7.5 years	NR	Statin use during RT was associated with significantly lower stroke incidence (adjusted HR 0.70, 95% CI 0.54–0.92, p = 0.01).Dose–response trend was noted
Miller (2024) [16]	USA	Retrospective cohort	Esophageal cancer	238	Baseline cardio-preventive medications (statins, aspirin, beta blockers)*Use recorded at diagnosis*	Investigate incidence of atrial fibrillation and cardiovascular events after esophageal RT	AFMACE (AF, heart failure, ventricular arrythmia, sudden death)	NR	NR	1.9 years	NR	21% of patients developed new AF and 33% had MACE after RT Higher left atrial radiation dose was strongly predictive of AF/MACE

*HNC* Head and Neck Cancer, *RT* Radiotherapy, *TIA* Transient Ischemic Attack, *MI* Myocardial infarction, Non-Small Cell Lung Cancer, *NR* Not reported, *AHA* American Heart Association, USPSTF (US Preventative Services Task Force), *AF* Atrial Fibrillation

### Cardioprotective medications and risk of cardiovascular and cerebrovascular events

#### Risk of cerebrovascular events

**Fig. 2:**
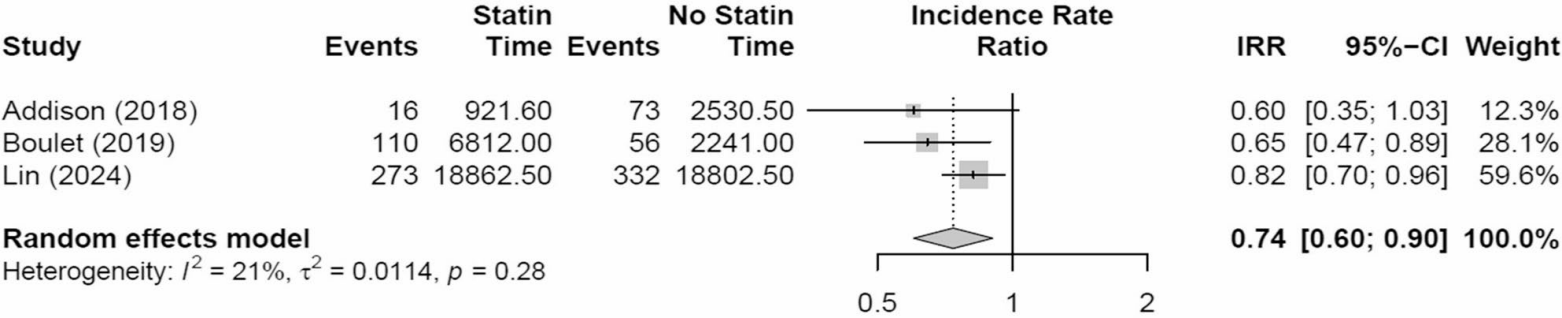
Effect of statin treatments on cerebrovascular events

Meta-analysis of three studies suggested that patients treated with radiotherapy had a reduced risk of cerebrovascular events (stroke) with incidental statin use (IRR 0.74, 95% CI 0.60 to 0.90, *p* = 0.034, I^2^ = 21%, τ^2^ = 0.011, Fig. 2). There was variability in tumor sites between the studies, with the study by Adison et al.[12] including patients with all types of head and neck cancer, whilst the study by Lin et al.[15] included patients with nasopharyngeal cancer only. Conversely, the study by Boulet et al.[20] included patients with either head and neck cancer (12%) or cancers of the chest or thoracic region (88%).

#### Cardiovascular events

**Fig. 3:**
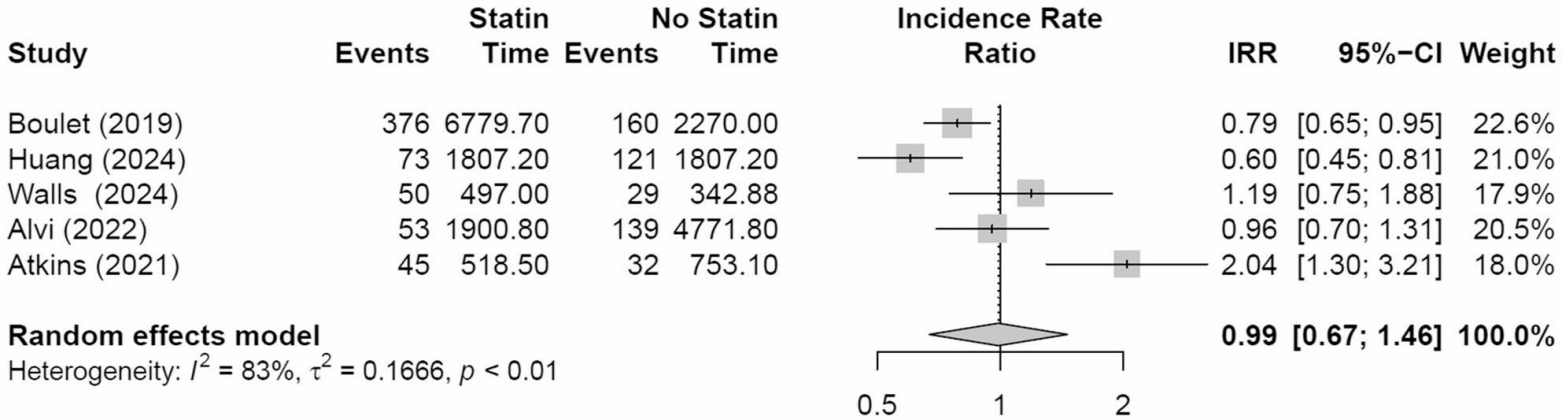
Effect of statin treatments on major adverse cardiac events

There were five studies which investigated the effect of statin therapy on major adverse cardiovascular events (MACE) for patients receiving RT to the thorax. There was no evidence that cardioprotective medications reduced the incidence of MACE in patients treated with RT (IRR 0.99, 95% CI 0.67 to 1.46, *p* = 0.961, I^2^ = 82.6%, τ^2^ = 0.167, Fig. 3). One study could not be included in the meta-analysis as it did not report the event rates for a statin and non-statin group but performed a univariate analysis which demonstrated no association between statin use and MACE (HR 1.38, 95% CI 0.89 to 2.15, *p*= 0.15)[16]. There was significant heterogeneity in tumor sites with one study investigating patients with thoracic and head and neck cancers[20], one study investigating breast cancer[19], two studies investigating lung cancer [17, 18] and one study with patients with head and neck cancer [10]. Only one study included in the MACE meta-analysis [19] used statistical methods to account for baseline differences in demographics between the cardioprotective and no cardioprotective groups.

### Proportion of patients with indications for cardioprotective medications

**Fig. 4:**
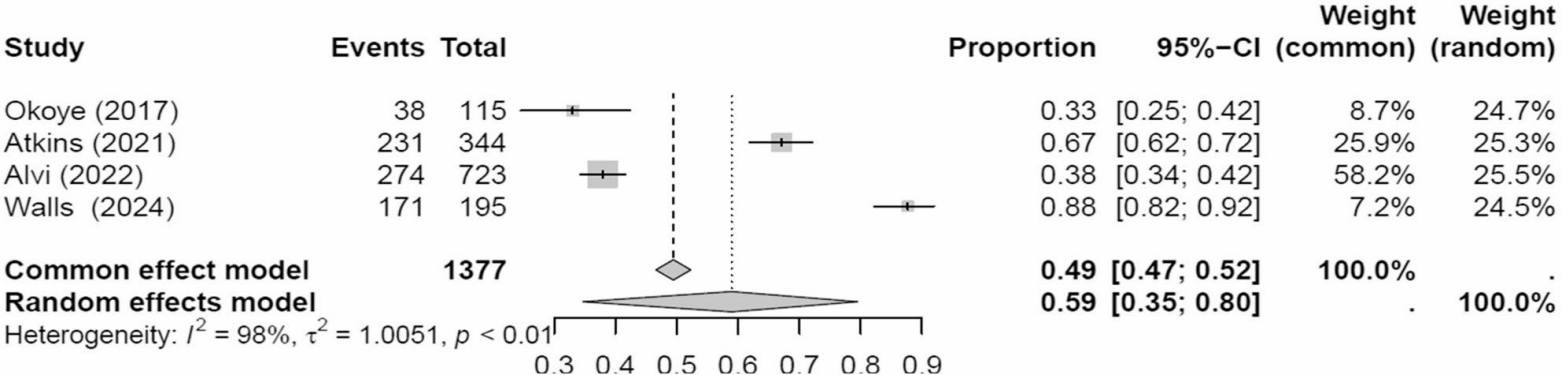
Proportion of patients not using statin treatments with indications to commence

A meta-analysis of four studies suggested that 59% (95% CI 35% to 80%, Fig. 4) of patients treated with RT not on statin therapy had established indications for commencement.

### Risk of bias assessment

All included studies were retrospective observational studies and were assessed using the Newcastle Ottawa Scale (Table 2). The quality of included studies was variable, with three studies scoring 9 out of 9 [14, 15, 19], whilst six studies were between 6 and 7 [12, 13, 16–18, 20]. One study was rated 3 out of 3 as it had no comparator group but was still eligible for inclusion as it reported the proportion of patients treated with RT who were not on statin therapy but had indications to commence them [21]. There were only 4 studies which followed patients up for greater than a median of 5 years [13–15, 19]. Only 3 studies used propensity score matching to balance the cohorts (cardioprotective medications compared to no medications) [14, 15, 19].

**Table 2:** Risk of bias assessment

Author	Addison et al. [12]	Alvi et al. [13]	Atkins et al. [17]	Boulet et al. [20]	Hsu et al. [14]	Huang et al. [19]	Lin et al. [15]	Miller et al. [16]	Okoye et al. [21]	Walls et al. [18]
*Year Published*	2017	2022	2021	2019	2016	2024	2024	2024	2016	2023
Selection										
1. Representativeness of exposed cohort Representative of the average patient receiving cardioprotective medications in the community	* All RT patients from one institution, somewhat representative	* Head & Neck cancer patients from one centre, broadly representative	* Patients with locally advanced NSCLC treated with thoracic RT at a tertiary centre between 1998 and 2014	* Population-based provincial database of patients > 65 yrs who had prior cardiac evaluation and had been treated with RT for head and neck or thorax cancer	* National wide Taiwanese cohort of HNC patients treated with RT between 1998 and 2008	* National wide Taiwanese cohort of left-sided breast cancer patients treated with RT from 2016–2019	* National wide Taiwanese cohort of NPC patients that underwent CCRT from 2012–2018	* Oesophageal cancer patients treated with RT from 2007–2019 at a single institution.	* HNC patients treated with curative RT from 2011–2013 at a university cancer centre; typical of tertiary care.	* Consecutive NSCLC patients treated with curative-intent radiotherapy between 2015–2020 at a single regional centre.
2. Selection of non-exposed cohort Drawn from the same community as the exposed cohort?	* Controls were same hospital RT patients without statins	* Non-statin group drawn from same institutional cohort	* Non-statin patients from same treated cohort	* Non-statin group from same Quebec cohort	* Non-Oral Antithrombotic Therapy users from same national cohort	* Non-statin patients from same national cancer registry database	* Non-statin group from same national NPC cohort	* Patients not taking cardioprotective medications in the same cohort	N/A	* Non-statin group from same hospital population and timeframe
3. Ascertainment of exposure (how was it recorded who had cardioprotective medications?)	* Statin use documented via EMR medication list	* Statin use, and eligibility determined from cohort and risk score criteria	* Statin use at RT start confirmed from medication lists or prescriptions in medical record	* Statin use from provincial pharmacy claims	* Anti-thrombotic use obtained from insurance prescription records.	* Statin use obtained from National Health Insurance Research Database Pharmaceutical subsidies	* Statin use identified via National Health Insurance Research Database Pharmaceutical subsidies	* All baseline characteristics including cardioprotective medication use collected via manual chart review	* CVD risk factors and medications obtained from medical records at diagnosis	* Statin therapy and dose intensity abstracted from clinical records. Medication use relied on documented prescriptions rather than self-report.
4. Demonstration that outcome of interest was not present at start of study	- Included patients with prior stroke/TIA ~ 6.9% had CVE at baseline	- Patients with prior ASCVD were included - baseline CVD not excluded	- No exclusion of baseline CVD events	- No exclusion of baseline CVD events	* Excluded any patient with prior stroke/TIA before RT	* Excluded any prior MACE or cardiac event before RT	* Excluded patients with any prior stroke, carotid disease, or metastasis before RT	* Excluded patients with any prior atrial fibrillation; “incident AF” defined as new-onset post-RT	N/A	- There was no exclusion of baseline CVD events, so prevalent disease could have influenced outcomes.
Comparability										
1. Study cohorts are similar in regard to CVD risk factors	- Statin users had more risk factors; groups not initially comparable	- Statin eligible vs. non had inherent risk differences by definition	* Multivariable cox regression model adjusted for CVD risk factors	- Statin users had higher burden of risk factors and comorbidities	* Propensity score-matched analysis achieved no significant baseline differences	* Propensity-score matched; statin vs. non-statin groups well balanced across various CVD risk factors	* Inverse probability treatment weighting used; baseline characteristics balanced between statin vs. non-statin groups	- No predetermined groups similar in CVD risk factors	N/A	* Fine-Gray regression model for CVD events adjusted for cardiac risk factors
2. Study cohorts are similar in regard to age	* Multivariable Cox adjusted for age and CVD risk factors	- No separate age adjustment for eligibility groups; adjusted analysis performed for actual statin use	* Multivariable cox regression model adjusted for age, sex comorbidities	* Multivariate time-dependent Cox regression model adjusted for age, sex and baseline co-morbidities	* Cox regression models adjusted for age, sex, and comorbidities	* Multivariable cox regression models adjusted for age, sex, and comorbidities	* Multi-variable fine-gray model adjusted for age, sex, stage, smoking	* Multivariate predictors of MACE after RT adjusted for age, RT dose, diabetes, aspirin use, beta-blocker use	N/A	* Fine-Gray regression model for CVD events includes adjustment for age
Outcome										
1. Assessment of outcome	* Outcomes adjudicated by blinded specialists via records	* ASCVD events from records, adjudicated blinded to exposures	* MACE and deaths identified via chart review and adjudication by cardiologist	* Outcomes stroke, MI via linked hospital records and validated ICD codes.	* Stroke/TIA outcomes identified via claims and ICD codes assigned by physicians	* MACE outcomes captured via claims and national death registry, ensuring independent verification.	* Ischaemic stroke events captured via ICD-9/12 codes assigned by neurologists	* AF outcome assessed rigorously: cardiologists blinded to RT dose adjudicated AF from ECGs, monitors	* CVD diagnoses confirmed via history; risk calculated by Framingham Score	* Clinical records were interrogated for cardiac events with three cardiologists verifying cardiac events
2. Was follow-up long enough for outcomes to occur (minimum of 5 year)	-	*	-	-	*	*	*	-	N/A	-
What was the median duration of follow-up?	Median 3.4 years, ≈ 40 months	Median 6.6 years	Median 20.4 months follow-up; survivors median 48.1 months	Mean follow-up ~ 1.5 years; short due to data limits	Follow-up up to 11 years; broad range. Median not stated.	5.02 years	7.5 years	22.8 months	24 months	21.1 months
3. Adequacy of follow-up cohorts	* No losses reported; retrospective chart follow-up to last visit	* Complete outcome capture via linked databases; minimal loss	- Vital status obtained for all patients, but no statement on other losses	* No loss to follow-up; comprehensive provincial health data capture.	* No loss to follow-up; 99.9% population coverage in National Health Insurance Research Database	* Complete follow-up via linked registry	* Complete follow-up; national cancer registry with follow-up to end of 2020.	- High mortality of oesophageal cancer limited long-term follow-up	N/A	* Good data availability, patient attrition was rare.
Total (out of 9)	6	6	6	6	9	9	9	6	3/3	7

## Discussion

This systematic review demonstrates the paucity of evidence exploring cardioprotective medication use in patients treated with RT. Most included studies were retrospective, with no randomized control studies conducted to date. Many patients treated with RT have indications for the commencement of statins although are not currently prescribed them.

Whilst this systematic review and meta-analysis suggests that the risk of cerebrovascular events (stroke and TIA) is reduced with statin therapy in patients treated with RT, this finding must be interpreted with caution given the small number of studies in the meta-analysis and the significant between study heterogeneity. All three studies investigating statin use in the HNC population were retrospective in nature and were investigating incidental statin use which introduces inherent biases. Two of the three studies included in the cerebrovascular meta-analysis [12, 20] were of moderate quality (both 6 out of 9 on the Newcastle Ottawa Scale), suggesting that the overall strength of evidence is low. Both these studies [12, 20] did not balance for confounders between the two groups (statin and no statin groups) and they had short follow-up periods (less than 5 years). Addison et al. reported a reduction in the risk of CVE (stroke or TIA) post RT for patients on statin therapy in a multivariable regression analysis (HR 0.41, 95% CI 0.21 to 0.80) [12]. Similarly, Boulet et al. observed a 32% reduction in stroke incidence among post-RT statin users (aHR = 0.68, 95% CI 0.48–0.98) for patients treated with RT to the head and neck or thorax [20]. Utilizing a cohort of patients with nasopharyngeal cancer only, Lin et al. suggested that statin use during RT was associated with reduced risk of ischemic stroke (aHR 0.70, 95% CI 0.54 to 0.92, *p*< 0.01) [15]. Randomized controlled trials examining the effects of cardioprotective medications, specifically statins, in a population of patients treated with RT would be of high clinical significance. Many important clinical questions remain, such as whether statin therapy needs to be initiated during or after radiotherapy, or whether patients without traditional CVD risk factors but are receiving radiotherapy would benefit from the early introduction of statin therapy. For patients with human papillomavirus associated oropharyngeal cancer or nasopharyngeal cancer this is especially important, as these patients commonly are younger, have improved survival and are less likely to have other pre-existing co-morbidities.

The evidence for the role of cardioprotective medications in reducing risk of major adverse cardiovascular events (MACE) for patients receiving RT to the thorax is more heterogeneous, with this meta-analysis suggesting no benefit. However, this is a varied group of studies comprising different sub-groups of patients. There was only one study which demonstrated a benefit to cardioprotective medications in this population and this was for patients treated with adjuvant RT for breast cancer [19]. This manuscript by Huang et al. suggested statin therapy more than halved the 5-year MACE incidence (12.2% vs. 31.7% in non-statin users), with a hazard ratio of 0.34 (95% CI 0.25–0.44) favoring statin users [19]. In patients with lung cancer, Atkins et al. found that statin users and non-statin users had similar rates of cardiac events (adjusted HR 1.18, *P*= 0.69) [17]. Walls et al. suggested that patients receiving statin therapy had improved overall survival, however there were no differences in cardiac events between the two groups [18]. 

Another area of ongoing research is whether the intensity of statin therapy is correlated with cerebrovascular or cardiovascular outcomes in patients treated with RT. Three studies identified in this systematic review investigated the effect of statin intensity, one study in patients with lung cancer [18] one in patients with nasopharyngeal cancer [15] and one in patients who were treated for breast cancer [19]. All three of these studies suggested that there was a dose response relationship for statin therapy. The study by Walls et al.[18] demonstrated that patients receiving high-intensity statin therapy had improved survival. Similar results were demonstrated in the study by Lin et al.,[15] with higher intensity statin therapy associated with a lower hazard ratio of ischemic stroke. The timing of statin therapy is another issue which requires ongoing investigation. This systematic review identified that definitions of statin use were heterogenous (Table 1) with some studies defining use as during or at the time of RT, and others defining statin users as those who were prescribed statins at any time after RT. Future studies to address whether the timing of statin use in relation to RT impacts cerebrovascular or cardiovascular even rates would be of clinical importance.

One of the major findings from this study is that many patients undergoing RT are not prescribed cardio preventative medications, with up to 60% of patients not on statin treatments having an indication to commence one. Whilst there is currently limited high-quality evidence to guide management options in patients treated with RT, it seems plausible that at a minimum clinicians should advocate for commencement of these cardioprotective medications as suggested in existing guidelines. There is also emerging evidence that the risk of CVD in patients treated with RT is higher than predicted by traditional risk scores. For example, the study by Miller et al. highlights RT as a risk factor for the development of arrythmias in a cohort of patients with esophageal cancer, with 21.4% of the cohort developing new-onset AF post-RT, 9-fold higher than predicted by Framingham scores [16]. The multi-disciplinary expert consensus statement from the International Cardio-Oncology Society [22] provides practical guidance to clinicians on the optimal identification and management of CVD risk in this population. For patients previously treated with RT, the recommendation is for regular screening and aggressive treatment of CVD risk factors and disease, although the time intervals for screening are dependent upon individual patient risk.

A major limitation of this systematic review and meta-analysis is the between study heterogeneity with variations in patient populations, tumor subsites and cardioprotective medication use definitions (either before, during or after RT). These heterogeneities limit the interpretability of the meta-analyses findings and suggest that further high quality prospective research is required to determine the impacts of statins in patients treated with RT. Another limitation is that the evidence base for cardioprotective therapies in patients treated with RT is uneven, with most eligible studies evaluating statins and limited data for antihypertensive or antithrombotic therapies. Furthermore, the protocol was not prospectively registered (PROSPERO), however, eligibility criteria, outcomes of interest, and analytic methods were defined prior to full-text screening, and PRISMA reporting was followed.

## Conclusion

Many patients undergoing RT are not prescribed cardioprotective medications as suggested by existing CVD guidelines. Future prospective research is needed to explore the impacts of cardioprotective medications specifically in patients treated with RT

## Supplementary Information

Supplementary Material 1.

## Data Availability

No datasets were generated or analysed during the current study.
