# Supplementary material for: Cardioprotective medications and the incidence of cardiovascular events in patients treated with radiotherapy: a systematic review and meta-analysis
Source: Cardiooncology. 2026 Jan 8;12:22. doi: 10.1186/s40959-025-00439-x (PMC12882525; doi:10.1186/s40959-025-00439-x)
Supplement: Supplementary file 1 — Supplementary Material 1. [file 40959_2025_439_MOESM1_ESM.docx]

Search Strategies

PubMed

("cancer" OR "malignancy" OR "carcinoma" OR "neoplasm" OR "tumour" OR "tumor") AND ("radiation therapy" OR "radiotherapy" OR "irradiation") AND ("statin" OR "lipid lowering" OR "cholesterol lowering" OR "antihypertensive" OR "anti-hypertensive" OR "blood pressure medication" OR "anti-thrombotic" OR "antithrombotic" OR "antiplatelet" OR "anti-platelet" OR "aspirin" OR "cardiovascular medication")

Scopus

TITLE-ABS-KEY (("cancer" OR "malignancy" OR "carcinoma" OR "neoplasm" OR "tumour" OR "tumor") AND ("radiation therapy" OR "radiotherapy" OR "irradiation") AND ("statin" OR "lipid lowering" OR "cholesterol lowering" OR "anti hypertensive" OR "anti-hypertensive" OR "blood pressure medication" OR "anti-thrombotic" OR "antithrombotic" OR "antiplatelet" OR "anti-platelet" OR "aspirin" OR "cardiovascular medication"))

Embase

('cancer':ti,ab,kw OR 'malignancy':ti,ab,kw OR 'carcinoma':ti,ab,kw OR 'neoplasm':ti,ab,kw OR 'tumour':ti,ab,kw OR 'tumor':ti,ab,kw) AND ('radiation therapy':ti,ab,kw OR 'radiotherapy:':ti,ab,kw OR 'irradiation':ti,ab,kw) AND ('statin':ti,ab,kw OR 'lipid lowering':ti,ab,kw OR 'cholesterol lowering':ti,ab,kw OR 'anti hypertensive':ti,ab,kw OR 'anti-hypertensive':ti,ab,kw OR 'blood pressure medication':ti,ab,kw OR 'anti-thrombotic':ti,ab,kw OR 'antithrombotic':ti,ab,kw OR 'antiplatelet':ti,ab,kw OR 'anti-platelet':ti,ab,kw OR 'aspirin':ti,ab,kw OR 'cardiovascular medication':ti,ab,kw)
